# Decision making on unsafe abortions in Sri Lanka: a case-control study

**DOI:** 10.1186/1742-4755-11-91

**Published:** 2014-12-17

**Authors:** Carukshi Arambepola, Lalini C Rajapaksa

**Affiliations:** Department of Community Medicine, University of Colombo, Kynsey Road, Colombo 8, Sri Lanka; Former Professor in Community Medicine, University of Colombo, Kynsey Road, Colombo 8, Sri Lanka

**Keywords:** Decision making, Unsafe abortion, Circumstances, Access and availability

## Abstract

**Background:**

Following an unintended pregnancy, not every woman would invariably choose to undergo an unsafe abortion. It suggests that in the decision making process, women face both ‘push’ factors that favour abortion and ‘pull’ factors that work against it. This study assessed the circumstances that surrounded a woman’s decision to undergo an unsafe abortion, compared to a decision to continue, when faced with an unintended pregnancy in Sri Lanka.

**Methods:**

An unmatched case-control study was conducted among 171 women admitted to nine hospitals in eight districts following an unsafe abortion (Cases) and 600 women admitted to the same hospitals for delivery of an unintended term pregnancy (Controls). Interviewer-administered-questionnaires and in-depth interviews assessed women’s characteristics, decision making process and underlying reasons for their decision. The risk of abortion related to their decision making was assessed using odds ratio (OR) and 95% confidence interval (CI).

**Results:**

Compared to controls, the cases were significantly less-educated, employed, unmarried and primi-gravid (p < 0.05). All knew the ‘illegal’ status of abortion, mainly through media (65.5% cases versus 80% controls). When making a decision, the risk of undergoing an unsafe abortion was significant among those who sought assistance (44% versus 32%; OR = 1.7 (95% CI = 1.2-2.4)), with more reliance placed on non-medical sources such as spouse/partner, friend, neighbour and family/relation. Speaking to women with past experience of induced abortions (31% versus 21.5%; OR = 1.6 (1.1-2.4) and failure in making the final decision with partners also imparted a significant risk for abortion (64% versus 34%; OR = 3.4; 2.4-4.8). A decision favouring unsafe abortion was predominantly based on their economic instability (29.5%) and poor support by partners (14%), whereas a decision against it was based on ethical considerations (44% religious beliefs: 12% social stigma) over its legal implications (4%). Most abortions were performed by unqualified persons (36.1% self proclaimed abortionists; 26.2% not revealed their qualifications) for a wide range of payment in non-sterile environments (45.9% unknown place) using septic procedures (38.5% trans-vaginal insertions; 24.6% unaware of the procedure).

**Conclusions:**

Women’s risk of unsafe abortion was associated with unreliable sources of information during decision making that led to poor knowledge and positive attitudes on its safety; poor access to affordable abortion services; and their economic instability.

## Introduction

In 1995, 45.5 million abortions that took place worldwide were considered illegally induced. Concerned by this trend, many countries liberalized their laws to increase the women's access to safe abortion. By 2003, illegally induced abortions were reduced to 42 million in the world. This declining trend of induced abortions was more or less similar in the developed countries, where abortion was legal and easily accessible, and developing countries, where it was predominantly illegal and restricted. However, there was a striking difference noted in relation to their safety, with 55% of all induced abortions being ‘unsafe’ in the developing countries, compared to a much less proportion in the developed countries [1, 2].

An unsafe abortion is defined as ‘a procedure for terminating an unintended pregnancy either by individuals without the necessary skills or in an environment that does not conform to minimum medical standards or both’ [3]. It leads to acute life-threatening as well as long-term disabling morbidity. Approximately 68,000 die from complications following unsafe abortions each year, giving a median mortality ratio of 34 per 100,000 live births in countries where abortion is illegal [4]. Consequences of such abortions are crucial in Asia, where it is concentrated among the poor, with a higher tendency towards severe complications [1]. It emphasises the need to prevent unsafe abortions among women facing an unintended pregnancy.

In Sri Lanka – a country in South Asia, with the expansion of its national family planning programme, a steady increase in the use of contraceptives has been noted from 32% in 1975 to 68% by 2006-7 among women in the reproductive age [5]. Despite this success, induced abortion remains a commonly practised fertility control method at the rate of 45 per 1000 women in the reproductive age (95% CI: 38-52) [6]. This rate is an indication of its growing demand although abortion is illegal in Sri Lanka. Despite being the most easily preventable cause [7], unsafe abortions is contributing considerably to the maternal morbidity and mortality in Sri Lanka, without showing a declining trend over the past few years [5]. In this backdrop, providing targeted interventions to women vulnerable to unsafe abortions would be of immense value.

Women perceive that many factors related to their socio-economic circumstances, fertility intentions, knowledge and attitudes on abortion, partner preferences, and availability and access to abortion services 'push' them towards an abortion [8–11]. These factors are population-specific and vary based on the legal, cultural and socio-economic background of a country. In Sri Lanka, past studies have implied poverty, poor knowledge and varying attitudes on abortion among women who seek abortion [12–14]. However, these findings may not be applicable in today’s context, since the fertility aspirations of modern Asian women have drastically changed over time.

Worldwide, 25% of unintended pregnancies end up as ‘unwanted or mistimed child births’ [15], highlighting that not every woman would invariably opt to undergo an abortion. More importantly, it further suggests that in addition to many ‘push’ factors that favour abortion, women also have ‘pull’ factors that work against abortion. All these factors during the decision making would determine a woman’s vulnerability to abortion. Although many factors favouring induced abortion have been identified among abortion seekers [16], their risk compared to women who decide against an induced abortion has not been evaluated. Identifying the risk of these factors would empower women to make the right choice through modification of the circumstances under which they make their decisions. This would be most crucial especially for women who would decide to undergo an unsafe abortion. Thus, this study intended to assess the risk of circumstances that surrounded women who decided to undergo an unsafe abortion, compared to women who decided to carry an unintended pregnancy to term.

## Methods

We carried out an unmatched case-control study including a qualitative component in nine hospitals in eight out of the 24 districts in Sri Lanka. Five of these hospitals were selected based on the highest frequency of all types of abortions reported in the Indoor Morbidity and Mortality Registers for each district [Medical Statistics Unit, unpublished]. Two hospitals were intentionally selected to ensure the representation of Muslim and estate-sector-Tamil populations. In the district of Colombo, both apex-referral tertiary hospitals for women were included.

Cases were women in the selected hospitals with complications following an unsafe abortion. Controls were mothers in postnatal wards following the delivery of an unintended pregnancy carried to term. The required minimum sample was based on 80% power to detect potential associations between the cases and controls at 5% alpha error; 20% minimum probability of exposure in the controls; odds ratio (OR) of 2; and 1:4 unmatched case-control ratio.

During recruitment, all women admitted to the gynaecology and medical/surgical casualty wards were screened consecutively over a period of six months for signs and symptoms suggestive of an abortion (e.g. period of amenorrhoea accompanied by vaginal bleeding, discharge, abdominal pain, fever or irregular menstruation). Of them, the potential ‘cases’ were identified by a confirmed diagnosis of ‘induced abortion’ based on the World Health Organization (WHO) criteria [17] under three categories: ‘certainly induced’, ‘probably induced’ and ‘possibly induced’ abortion. Of these women, all ‘certainly induced’ abortion cases (N = 122) and only the women who showed definitive clinical signs of infection plus received intravenous antibiotic treatment in the ‘probably induced’ abortion category (N = 49) were recruited as ‘cases’ for the study. This restriction ensured that the group of cases represented only the women who underwent an unsafe abortion.

For comparison, controls were selected during the same study period using a systematic sampling method among mothers admitted to postnatal wards of the same hospitals following the delivery of an unintended pregnancy carried to term. ‘Unintended pregnancy’ was identified using an interviewer-administered-questionnaire based on the WHO definition of ‘any pregnancy of a woman contracepting during the cycle of conception or not contracepting due to reasons other than desired pregnancy' [17].

Core questionnaire developed by the WHO for multi-centre hospital-based descriptive studies on abortions [17] was used for data collection. It was modified to suit the local conditions and its judgmental validity (face, content and consensual) was assessed by a panel of experts in maternal health care, public health and clinical psychology. It was administered by pre-intern medical officers who were not involved in providing care in the ward. Prior to data collection, they were trained by a group of psychologists and experts in qualitative research. Using the validated questionnaire, data were collected on demographic, socio-economic and reproductive characteristics, and on decision making (the process and reasons leading to pregnancy outcomes). Circumstantial details on pregnancy termination were obtained only from the ‘certainly induced’ abortion cases (N = 122). Furthermore, in-depth interviews were conducted by the principal investigator in a sub-sample of women (N = 13) to explore their decision making process. This sample size was decided by the point of saturation at which no new information was generated. Ethics clearance was obtained for the study from the Ethics Review Committee of the Faculty of Medicine, University of Colombo.

Data were analysed using Statistical Package for Social Sciences (SPSS)-Version 20. Descriptive statistics included proportions calculated for categorical data and mean and standard deviation for quantitative data. The risk of abortion associated with women’s socio-economic characteristics and their decision making was assessed by comparing the cases and controls using odds ratio (OR) and 95% confidence intervals (CI).

## Results

Of the 822 potentially induced abortions that were initially identified, 171 were recruited as cases for the study. The controls consisted of 600 post-partum mothers. There were two controls who had previously undergone an induced abortion and two cases who had done it for the second time.

The majority of women in both groups belonged to 25-29 age group, were married and of Sinhala-Buddhist origin. Compared to the controls, the significant characteristics of cases were being unmarried, primigravid, not having an informed decision on family size, less-educated, employed, having children and having longer last birth intervals (Table 1). Of the employed, more cases were in elementary occupations as manual labourers (39% cases versus 36% controls) or as plant/machine operators (34% versus 16%). The non-primi gravid women were at significant risk for unsafe abortion, if they had longer average birth intervals (3.4 vs. 2.9 years) and longer last birth intervals (5.7 vs. 4.8 years).

**Table 1:** **Risk of unsafe abortion in relation to the socio**-**economic status of women at the time of unintended pregnancy**

	Cases		Controls		Odds ratio
Characteristic	N = 171*		N = 600*		(95% CI)
	No.	%	No.	%	
**Current marital status**					
Single/divorce/separate/widow	31	18.1%	10	1.7%	**12.9 (6.3-27)**
**Parity (P)**					
Primi	36	21.1%	83	13.8%	**1.7 (1.1-2.6)**
**Decided on family size**					
No	89	52.0%	173	28.8%	**2.7 (1.9-3.8)**
**On contraceptives**					
No	103	60.2%	238	39.7%	1.5 (0.9-2.2)
**Living children**					
Yes	40	23.4%	92	15.3%	1.7 (1.1-2.6)
**Secondary education**					
Not completed	115	67.6%	332	55.5%	**1.7 (1.2-2.4)**
**Currently employed**					
Yes	69	40.4%	149	24.8%	**2.1 (1.4-2.9)**
**Type of occupation** ^**1**^					
Unskilled/Less skilled	52	76.5%	80	56.3%	**2.5 (1.3-4.8)**
**Secondary education of partner**					
Not completed	89	59.7%	308	52.1%	1.4 (0.7-1.6)

*In some variables, row values do not add up to the total cases and controls due to missing data.

^1^Includes only the employed women.

CI = confidence interval.

Nearly 50% of the partners of both cases and controls had completed their upper secondary education, while nearly all were in employment. Cases differed significantly from their partners who were more educated and employed in better-skilled occupations than them (p < 0.05). No such difference was seen between the controls and their partners.

Prior to their unintended pregnancy, all women in both groups had heard about induced abortion ‘as a method of pregnancy termination’ and its ‘illegal’ status. It was mainly through television and women newspapers (65.5% cases versus 80% controls) and to a lesser extent from immediate associates such as friends (14.6% versus 4.5%), neighbours (9.9% versus 11.7%) and family/relations (5.9% versus 5%).

When arriving at a decision about their pregnancy (Table 2), compared to the controls, a significant proportion of cases sought advice from others (44% cases versus 32% controls; p <0.05), with more reliance placed on non-medical sources such as their spouse/partner, friend, neighbour and family/relation, and also from women who had previously undergone induced abortion (31% versus 21.5%; p < 0.05). In the qualitative enquiry, cases revealed that their unwillingness to approach qualified persons was due to the illegal status of abortion, while some admitted to have relied on abortion experiences of others since they had not received any formal education on abortion before.

**Table 2:** **Risk of unsafe abortion in relation to the decision making process among women with unintended pregnancies**

Decision making process	Cases		Controls		Odds ratio
	N = 171		N = 600		(95% CI)
	No.	%	No.	%	
**Advice sought at decision making**					
Yes*	76	44.4%	190	31.7%	**1.7 (1.2-2.4)**
• Friend	38	22.2%	26	4.3%	
• Family/Relation	30	17.5%	12	2.0%	
• Neighbour	6	3.5%	8	1.3%	
• Medical personnel	0	0.0%	10	1.7%	
• Spouse/ Partner	62	36.6%	171	28.5%	
**Final decision made with partner**					
No	109	63.7%	205	34.2%	**3.4 (2.4-4.8)**
**Spoken to a person who had experienced an abortion**					
Yes	53	31.0%	129	21.5%	**1.6 (1.1-2.4)**
**Will undergo abortion if any unintended pregnancy in future**					
Yes	17	9.9%	7	1.2%	**19.7 (7.8-50.1)**
Difficult to say	58	33.9%	211	35.2%	**2.2 (1.5-3.4)**

*Multiple responses; sources of information do not add to the total who took advice.

CI = confidence interval.

Although women relied on partners during decision making, a higher proportion of cases made their final decision on abortion all by themselves, compared to the controls (63.7% versus 34.2%).

Remarkably, 12.3% of the cases initially considered continuing with their pregnancy, while 42.7% of the controls considered undergoing an abortion. The commonest reason given by cases for terminating their pregnancy was not being economically stable (Table 3). Other reasons included poor support from the partner, too old for carrying a pregnancy and too short last birth interval. The commonest underlying reason given by controls for continuing with the pregnancy was due to religious beliefs that portrayed abortion as a ‘sin’. In 12% of controls, it was due to cultural constraints that portrayed abortion as a socially unacceptable event. Good support from the partner, being economically stable and not having a short last birth interval also helped them in their decision.

**Table 3:** Comparison of the underlying reasons for undergoing abortion/continuing with the pregnancy between the cases (N = 122)* and controls (N = 600)

Underlying reasons for undergoing induced abortion	Cases		Underlying reasons for continuing with pregnancy	Controls	
	No.	%^1^		No.	%^1^
Economically not stable	36	29.5%	Religious beliefs on abortion	266	44.3%
Partner advocates abortion	17	13.9%	Partner supports pregnancy	167	27.8%
Too old for a pregnancy	14	11.5%	Economically stable	74	12.3%
Last birth interval too short	13	10.7%	Social stigma attached to abortion	73	12.2%
Unmarried status/ Widowhood	10	8.2%	Last birth interval not too short	50	8.3%
Extra-marital affair	9	7.4%	Forced by health worker	28	4.7%
Plans to go for a job	6	4.9%	Illegal status of abortion	25	4.2%
Not ready for motherhood	5	4.1%	Did not know how to get it aborted	23	3.8%
Rape	5	4.1%	Forced by partner, family or friends	21	3.5%
Forced by partner	4	3.3 %	Others know I am pregnant	6	1.0%
Female foetus on scan	1	0.8%	After visualizing the foetus on scan	1	0.2%

*Women who admitted to have undergone an unsafe abortion were only included in the analysis.

^1^Multiple responses; percentages given out of the total in cases or controls.

After making their decision to abort, the majority of cases approached their partners and/or immediate associates to obtain more information about the persons/places available for pregnancy termination (60%) and to accompany them to abortionists (52%) (Table 4). It was revealed in the qualitative enquiry that in their absence, women depended on unknown sources such as taxi drivers for this purpose.

**Table 4:** **Characteristics of the termination of pregnancy among women who underwent certainly induced abortion (N = 122)** ^1^

Characteristic	No.	%
**Sought help on ways to terminate the pregnancy**		
Did not seek help	49	40.2%
Sought help		
• Husband/Partner	37	30.3%
• Family/Relative	10	8.2%
• Friend/ Neighbour	27	22.1%
• Medical person	1	0.8%
• Three wheel Taxi drivers	3	2.5%
**Person who did the abortion**		
Qualified doctor	25	20.5%
Traditional healer	3	2.5%
Pharmacist	2	1.6%
On her own without any help	13	10.7%
Other (Friend/Husband/Family member)	3	2.5%
Self proclaimed abortionists	44	36.1%
Ignorant of his status/No response^2^	32	26.2%
**Delay since termination to reach the hospital**		
Within a day	13	10.7%
2-4 days	26	21.3%
5-7 days	18	14.8%
More than one week	24	19.7%
Not sure/No response^2^	41	33.6%
**Method used for termination of pregnancy**		
Trans-vaginal insertion	47	38.5%
Injections	7	5.7%
Indigenous medicine and tonic	16	13.1%
Abdominal or trans-vaginal application of pressure	7	5.7%
Vacuum aspiration	9	7.4%
Dilatation and curettage	1	0.8%
Medicine (E.g. Misoprostol)	5	4.1%
Ignorant of the procedure/No response^2,3^	30	24.6%
**Place of abortion**		
Private hospital	8	6.6%
GP Practice	14	11.5%
Own home/Friend’s house	7	5.7%
Unknown place (E.g. Abortionist’s house, boutique)	56	45.9%
Ignorant of the place/No response^2^	37	30.3%
**Persons who accompanied the woman for the abortion**		
Husband/Partner	41	33.6%
Friend	10	8.2%
Sibling/relation	12	9.8%
None	21	17.2%
No response^2,4^	38	31.1%
**Total**	**122**	**100.0%**

^1^Women who admitted to have undergone an unsafe abortion were only included in the analysis.

^2^Includes women who were reluctant to reveal the information.

^3^Also includes respondents who were not sure of the method used.

^4^Also includes 13 women who attempted termination by themselves.

Only 11% of cases attempted methods of self-induction. 46% of cases visited the abortionist more than twice and 14% more than five times. In the majority of cases, septic procedures with no pain relief were performed during termination by non-qualified abortionists, for a wide range of payment of Rs. 1,000-30,000. In the qualitative inquiry, women reported that most places lacked sterile or proper equipment and were run without assistance in the back room of a boutique, own home or in the house of a relative or abortionist. None took place in government hospitals or clinics run by non-governmental organizations. The most commonly used methods were trans-vaginal insertion of rods and injections. The worst experience reported in the qualitative inquiry was one woman collapsing following the insertion of a castor oil plant stem into the vagina for a fee of Rs. 30,000. In response to future intentions, 14% of cases alleged that they would resort to abortion in the event of another unwanted pregnancy, while 47.5% were not sure of their decision.

## Discussion

This study sheds light on the decision making process and circumstances leading to an unsafe abortion, in comparison with that leading to a mistimed birth, in a setting where abortion is illegal. It should be noted that these findings are not applicable to women who succeeded a safely induced abortion, but to those who developed complications following an unsafe abortion. Thus, this study is unique, providing evidence on the women who are most vulnerable to maternal morbidity and mortality.

Health status of Sri Lanka is sustained by the Government policy of ‘free health for all’ that has been instrumental in achieving a high health literacy and health seeking behaviour, especially among the females [5]. Our findings however are contrary to the expected, highlighting deficiencies in the provision of information to women on the health implications of unsafe abortion and women's utilisation of unsafe abortion services. In comparison, previous studies have shown that women face difficulties in obtaining both information and services on abortion in low-resource and low-literacy settings, irrespective of the legal status of abortion [11, 14, 18].

### The role of access to information in the decision making

Our study shows that a decision to abort using unsafe techniques is significantly associated with the risk of women seeking access to information about its safety during the decision making process (44% cases versus 32% controls). Furthermore, in the absence of any formal channel providing awareness, cases seemed to rely more than their controls on non-medical sources for knowledge. Despite the free health service that ensures access to state-owned health facilities throughout the country [5], the reluctance of women to approach a healthcare worker in the event of an unintended pregnancy, was probably due to the illegal status of abortion in Sri Lanka. Stigma attached to abortion could also be a major deterrent [11, 18]. With the wide accessibility of mobile telecommunication services even in the most remote areas in Sri Lanka, exploring the possibility of establishing 24-hour help lines for reliable information at the community level would be of much value [19]. Abortion education through media did not play a significant role in the decision making in our study. Nevertheless, close monitoring of any sensationalising abortion news by media is highly recommended. Furthermore, to prevent the vulnerable groups being misinformed, women need to be empowered with knowledge on the safety of abortion from an early age. Adolescence would be the ideal time to approach them through educational opportunities such as school-based internet inquiry [20, 21]. Though sexual health is already a vital component in the school curriculum, there may be cultural barriers in discussing these topics at school. It is thus important to provide skill development for teachers.

Previous research has implied varying attitudes on unsafe abortion among women who seek abortion [1, 14, 18, 22]. Our study implies that the risk of unsafe abortion associated with access to unreliable non-medical sources of information is not only because it leads to poor knowledge on its safety but also because it formulates positive attitudes towards induction. Close associates seemed to play a crucial role in changing women’s perceptions on abortion as a 'safe' procedure. In particular, women showed a tendency to confide more in other women’s experience of successful inductions. This type of ‘herd behaviour’ was probably facilitated by their low socio-economic status characterised by low level of education and employment in unskilled/semi-skilled occupations. The equally low social status of their co-workers seems to reinforce the women’s poor access to correct information as well as to safer abortion services. It is shown that unlike home-bound women, employed women are more at risk for unsafe abortions, with many opportunities for clandestine sexual relationships and peer influences [23]. Therefore, multi-pronged interventions in the form of counselling services should take priority in these high-risk settings. With high literacy among women in Sri Lanka, work settings could be equipped with educational material and contraceptives. Evidence recommends behaviour change communication campaigns through inter-personal approaches that engage community leaders and influencers [18], dialogue-based groups that negotiate the social support they need when making decisions [24] and community intermediaries that create an enabling environment [25] as feasible methods.

In previous studies in Asia, a significant decision maker has been the partner with their direct involvement through "orders" to abort or indirectly through denial of responsibility for the pregnancy [11, 26]. Our findings are in contrast to this, highlighting the changing women status in Asia. Although partners of cases were of better socio-economic status than of their controls, partners’ influence seemed to be minimal when making the final decision to abort a pregnancy. However, once the decision was made, men were utilised more by cases to accompany them to abortionists.

### The role of safe abortion services in the decision making

Increased availability as well as accessibility have shown to improve the quality of induced abortions in countries where abortion is legalised [22, 27, 28]. Our study verifies that the vulnerability of Sri Lankan women to unsafe abortion is mainly due to poor access to affordable abortion services. This was explicit, with an increased risk of unsafe abortion seen among women of low socio-economic status. It is alleged that safe abortion services are not uncommon in the country, but almost always offered for a substantial fee. This is in contrast to the health seeking behaviour in the past, during which 'menstrual regulation methods' were offered to clients for an affordable fee by a few non-governmental organizations [29].

### Circumstances leading to unsafe abortion

Worldwide, the commonly reported reasons for unsafe abortion are postponement or completion of child-bearing and socio-economic concerns such as poverty and unemployment [10, 29, 30]. However, a recent study concluded that a decision to undergo abortion is typically motivated by multiple, diverse and inter-related reasons on financial constraints and lack of partner support [31]. These factors coincide well with our findings, which confirm that a decision favouring unsafe abortion was predominantly based on their economic instability and poor support given by partners.

Other than the 'push' factors, several 'pull' factors that prevent a decision of abortion were also identified in our study. Sri Lanka is a multi-ethnic country with strong religious convictions. In a local study among women, 94% felt that abortion was a ‘sin’ while 66% felt that it should be available on demand for married women [12]. It is interesting to note that women who decided against abortion considered more about the ethical aspect (44.3% religious beliefs: 12.2% social stigma) than its legal status. This implies that even if abortion is legalised, safe abortion services would remain under-utilised, unless the stigma attached to abortions that leads to the reluctance of both women accessing the services and the health care staff providing it, is addressed. Under-utilisation of safe abortion services due to poor knowledge [18], religious beliefs [32, 33] and stigma [14, 34] has been observed elsewhere. Holmgren & Uddenberg [32] have reported that women's main moral dilemma was not a conflict between the woman and her foetus, but a conflict between several close relationships, also concerning the prospective father [32]. It is imperative that health managers ensure that clients’ information against unauthorised disclosure is protected by creating a respectful environment, with physical space for assuring privacy, a wide-range of skills for building rapport with women in a culturally-attuned empathic manner and attitudinal changes at all levels of health care providers to treat them with dignity, so that women are comfortable in discussing their decision making with health staff. Sri Lanka Government health policy aims to facilitate equity through increased access to health services and quality of care.

### Methods of abortion

Circumstances under which most of the unsafe abortions took place illustrate the extremely poor quality of the services provided by Abortionists. Most were done by unqualified persons for a wide range of payment in unsterile environments using septic procedures. It is evident from our study that the procedures used by abortionists had been more or less similar, compared to the methods used on abortion cases in hospitals a few decades ago [29, 35]. Legal enforcement of severe punishment to these unqualified abortionists would be effective. This would prevent the less resourced districts to have access to unsafe abortion services. Decisions to refer or to keep in their care by abortionists are strongly associated with the patients' financial resources [36]. In our study too, women's health seeking behaviour following an unsafe abortion was determined by the advice provided by the abortionist; poorer women to visit the hospital immediately and others to be treated in a private hospital.

### Limitations

Our sample has not captured the women who sought care outside Government hospitals following post-abortion complications. This could have led to an over-representation of cases of poor socio-economic status, since the state owned health services are free of charge and thus, predominantly accessed by people of relatively low socio-economic status. However, this selection bias was minimised by having controls accessing the same hospital services s as the cases.

## Conclusions

A decision favouring unsafe abortion was predominantly based on their economic instability and poor support given by partners, whereas a decision against it was based on ethical considerations over its legal implications. Reliance on non-medical sources of information such as immediate associates leading to poor knowledge as well as positive attitudes on its safety played a crucial role in the decision making process towards an unsafe abortion. Unsafe methods of termination used have not changed over time. It is believed that the findings of this study would also be applicable to similar settings in developing countries.
